# Supplementary material for: Cell motility empowers bacterial contact weapons
Source: ISME J. 2024 Jul 29;18(1):wrae141. doi: 10.1093/ismejo/wrae141 (PMC11482024; doi:10.1093/ismejo/wrae141)
Supplement: BoothMeacockFoster_2024_ISME-revision_Supplementary_Figures_wrae141 [file boothmeacockfoster_2024_isme-revision_supplementary_figures_wrae141.pdf]

Supplementary Table 1 | Primers used for construction of Δ*pilU* strains and q-RT-PCR

Primer	Use	Sequence
pilU_Up_F	*pilU* upstream region	CAAGCTTCTGCAGGTCGACTCTAGAGGATCgcagaccctgatcaagaagatcg
pilU_Up_R	*pilU* upstream region	gcctactgaagacggttcagcggaagcgccattccatgatgttctcgctcactc
pilU_Down_F	*pilU* downstream region	gcccctgagtgagcgagaacatcatggaatggcgcttccgctgaacc
pilU_Down_R	*pilU* downstream region	ACCCGTGGAAATTAATTAAGGTACCGAATTtcggcgtggccttctatatcc
acp_F	*acpP* qPCR	ACTCGGCGTGAAGGAAGAAG
acp_R	*acpP* qPCR	CGACGGTGTCAAGGGAGT
CDI1_F	CDI 1 qPCR	cgcgatgaaggcaacctgc
CDI1_R	CDI 1 qPCR	ccgtggacgttcaactcg

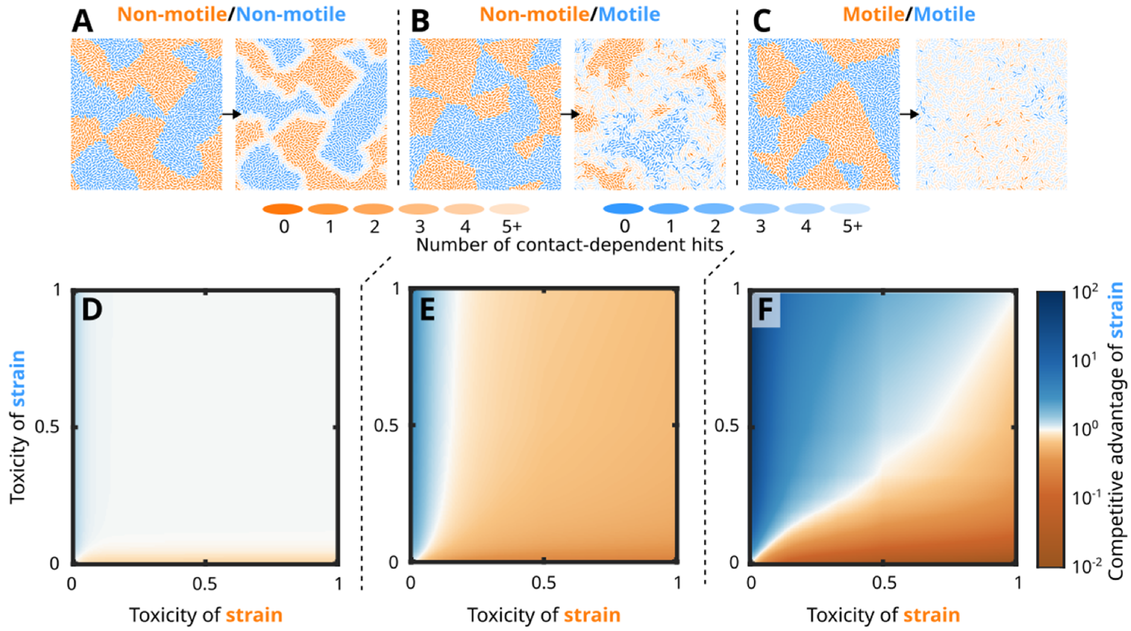

Figure S1 | Asymmetric motility influences competitive outcomes in the IBM. We simulated asymmetric motility scenarios representing, for example, inter-specific competition using the IBM. In **A**-**C** we show snapshots indicating the number of hits accumulated at $t = 500$ by two strains with equal firing rates of $\lambda = 0.02$ inoculated at equal starting densities. The motility of the two strains is either symmetric (non-motile, $F = 0$, **A**, and motile, $F = 1$, **C**) or asymmetric (motile mixed with non-motile, **B**). These hit distributions are then converted into competitive outcomes in **D**-**F** for each combination of possible toxin potencies ξ for the two strains. The overall strength of competition in the asymmetric case is intermediate between the two symmetric extremes, but also favours the non-motile strain for most toxicity combinations due to its ability to form protective clusters that resist invasion by its motile competitor.

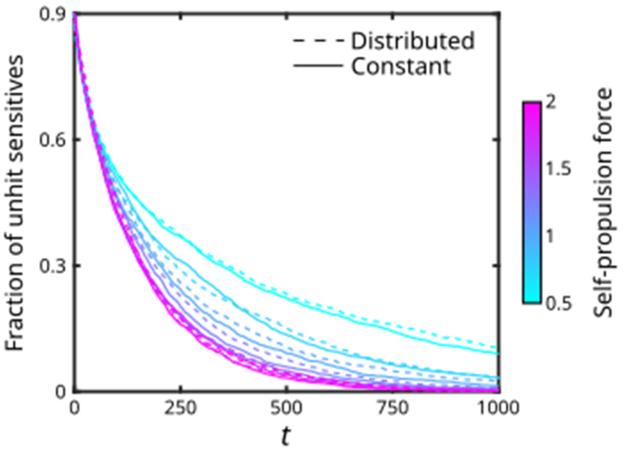

Figure S2 | Different microscopic firing mechanisms result in equivalent macroscopic killing dynamics. To investigate the importance of the mechanistic basis of different types of contact-dependent weapons for determining killing dynamics (Methods), we performed IBM simulations with different intoxification processes. In the 'Distributed' simulations, we assumed that surface-based toxins were produced stochastically at a rate λ and distributed equally between all cells currently contacted, representing a delivery mechanism such as CDI where the toxin can remain active on the cell surface until it is taken up by a neighbouring cell. In the 'Constant' simulations, all currently contacted cells were stochastically intoxified at a fixed rate λ, representing a toxin delivery mechanism such as the T6SS where delivery is only possible at the time of firing. We find that the overall intoxification dynamics are equivalent between the two mechanisms, provided the firing rate λ was rescaled by the average number of cell contacts $C = 5$ ($\lambda = 0.1$, Distributed, $\lambda = 0.02$, Constant). Simulations were initialised under homogeneous (non-patchy) starting conditions, with an initial fraction of attackers of 0.1.

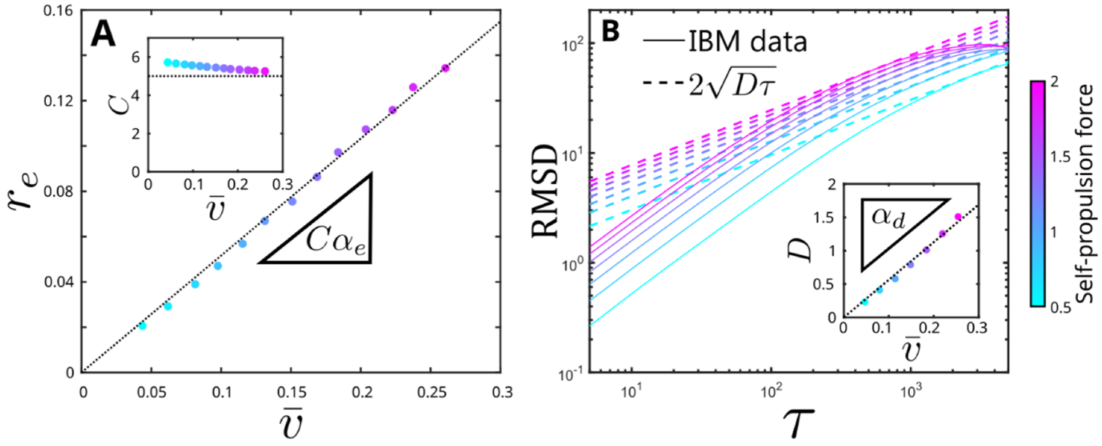

Figure S3 | Continuum model parameters can be extracted from the IBM. To find the relationships between average cell speed $\bar{v}$ and the target switching rate r_e and diffusion constant D associated with genotypic mixing, we extracted relevant statistics from self-propelled rod simulations run with different self-propulsion forces. (**A**) We directly measured the rate at which cells made contact with new individuals, yielding the per-cell target switching rate r_e. This is the product of the per-contact contact switching rate and the total number of cell contacts C, which is weakly dependent on system activity (inset), probably due to activity-dependent density fluctuations (62). We assume $C = 5$ (dotted line) throughout this manuscript – the closest integer value under most conditions – although we obtain substantially similar results for $C = 6$. (**B**) We further measured the diffusion constant of simulated cells by calculating their Root Mean Squared Displacement (RMSD). We estimate the effective diffusion constant D of cells at these long timescales by finding the tangent to the log-transformed RMSD data with a slope of 1/2 (dashed lines). In both **A** and **B**, we found a simple proportional relationship between $\bar{v}$ and the statistic in question. We fit the proportionality constants α_e and α_d to these, allowing us to objectively parameterise the connection between our IBM and continuum frameworks (Fig. 1).

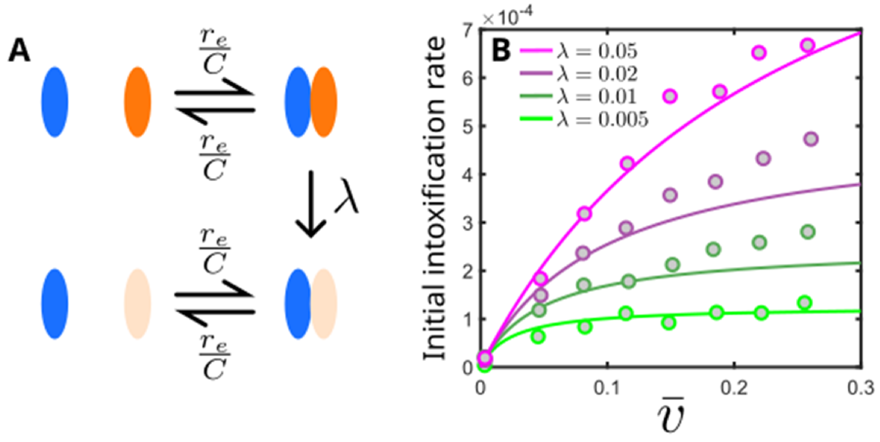

Figure S4 | Intoxification efficiency is a saturating function of system velocity under homogeneous starting conditions. (**A**) Under homogeneous (non-patchy) conditions, contact exchange is the sole velocity-dependent process limiting intoxification efficiency. We can model the resulting intoxification dynamics using an enzyme-like reaction scheme, with the per-cell contact exchange rate $r_{e=} C\alpha_e\bar{v}$, number of cell contacts C and weapon firing rate λ setting the various rates in the system. (**B**) From this, we can predict the initial intoxification rate of a small invading population of attackers as a function of the average cell velocity $\bar{v}$ and weapon firing rate λ (Supplementary Notes). Solid lines are predictions from the analytical model, points are IBM outputs.

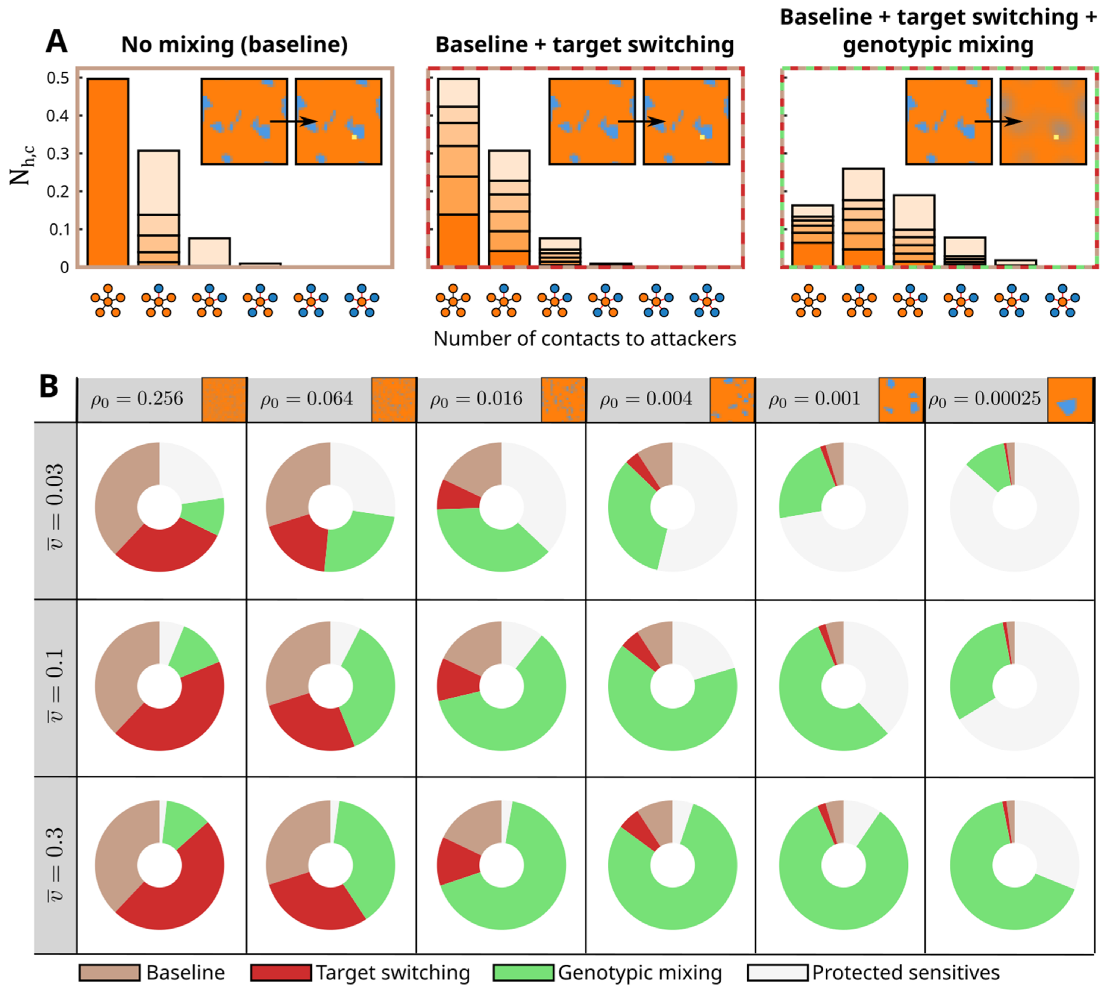

Figure S5 | The continuum framework allows us to disentangle contributions from the two forms of mixing. (**A**) From equivalent starting conditions, continuum simulations can be run with no mixing (left), only contact switching active (middle) or with both contact switching and genotypic mixing active (right). (**B**) By comparing the number of cells intoxified under each of these conditions, we can split the intoxification dynamics under a given set of conditions into a baseline component guaranteed by the starting conditions even in the absence of mixing (brown), a component mediated by contact switching (red) and a component mediated by genotypic mixing (green). In addition, a portion of the sensitive population remains unaffected even when both forms of mixing are active (light grey). Here we show the variable contributions of the two mixing processes when varying motility and patchiness. In (**A**), the contact distributions shown are taken at t = 1000 of a simulation run with the specified mixing types active. The lattice site sampled to generate these contact distributions is highlighted with a yellow square in the insets. In all cases, simulations were run with an attacker fraction of 0.1.

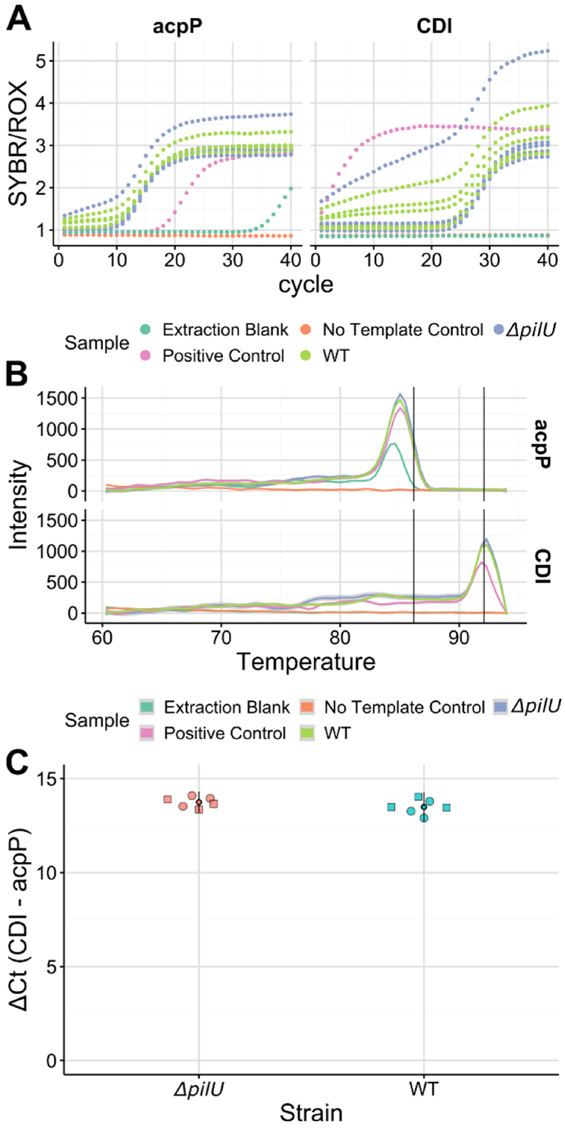

Figure S6 | CDI expression is not altered in pilus mutants. Quantitative reverse-transcriptase (qRT-PCR) of RNA extracted from colonies of wild-type and ΔpilU strains of *Pseudomonas aeruginosa* PAO1 shows that expression of CDI 1 (PA0040-PA0041) similar. **A** raw outputs (SYBR/ROX signal) for the house-keeping gene *acpP* and CDI, for samples including RNA extraction blank, qPCR control reaction with no template and gDNA (Positive Control). **B** Melting curves of products, with the predicted melting points (Tm) for *acpP* (86.2 C) and CDI (92.1 C). **C** ΔCt for wild-type and pilU deletion mutants. Each point is a single technical replicate qPCR reaction, squares and circles indicate distinct biological replicates. Data were analyzed in MXPro 4.1, see supplementary methods for details.

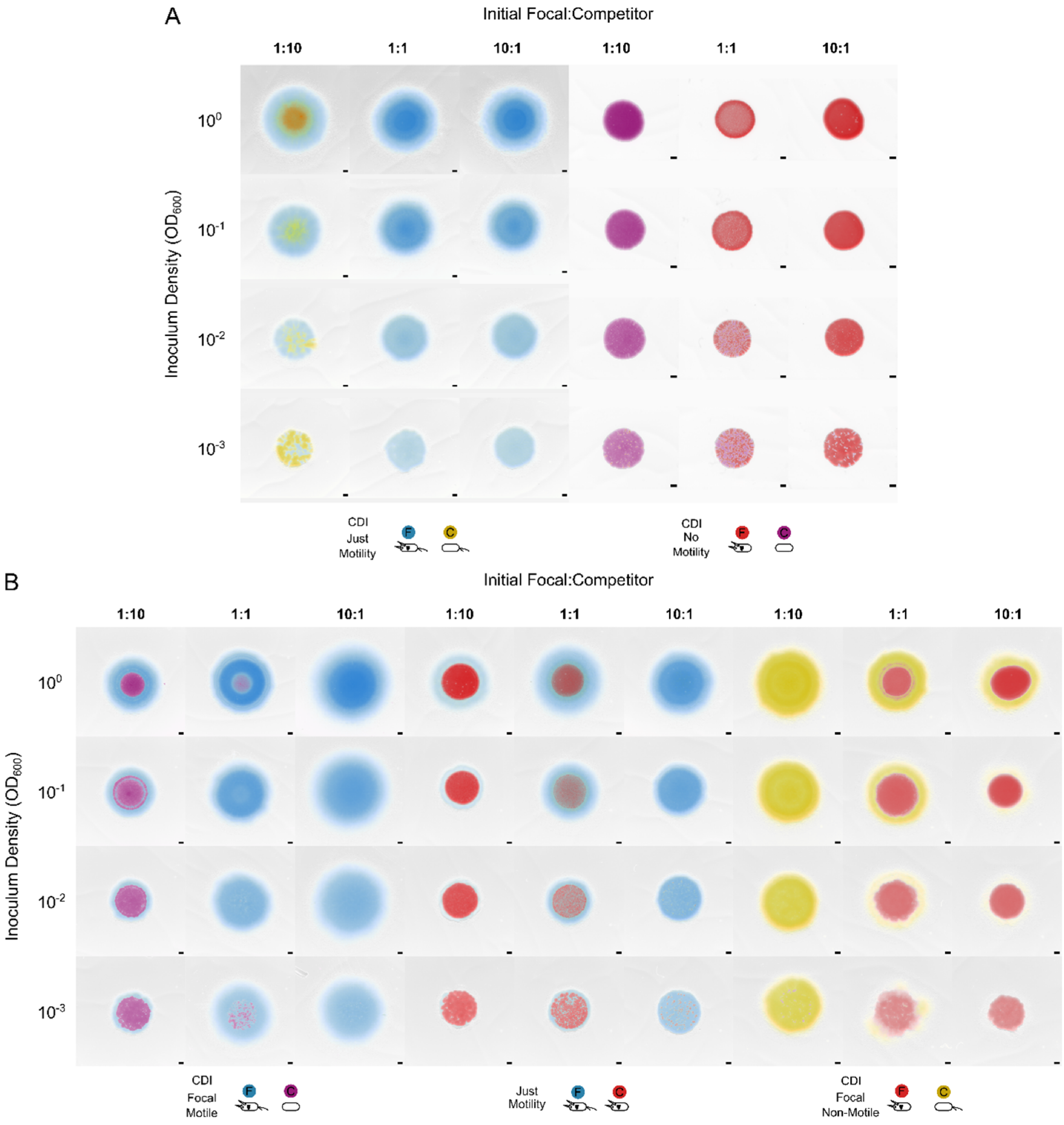

Figure S7 | Motility and CDI provide a competitive advantage at the center and edge of colonies regardless of the initial density and attacker frequency. Representative microscopy images from colony competitions of *P. aeruginosa* strains inoculated with different initial densities (OD_{600}) and ratios of focal (F) to competitor (C) after 48 h of growth show differences in the scale and structure of communities. (**A**) Competition between focal strains armed with CDI against a sensitive competitor with active motility (left) and motility inactivated (right). (**B**) Competition between a CDI armed motile focal strain and non-motile sensitive competitor (left), between a motile focal strain and non-motile competitor (center) showing the effect of just motility (no CDI sensitivity in the competitor strain), and between a focal non-motile CDI armed strain and a motile CDI sensitive competitor (right). Images are false-coloured: blue (motile CDI armed), red (non-motile CDI armed), yellow (motile CDI sensitive) or purple (non-motile CDI sensitive. Scale bar: 500 µm.

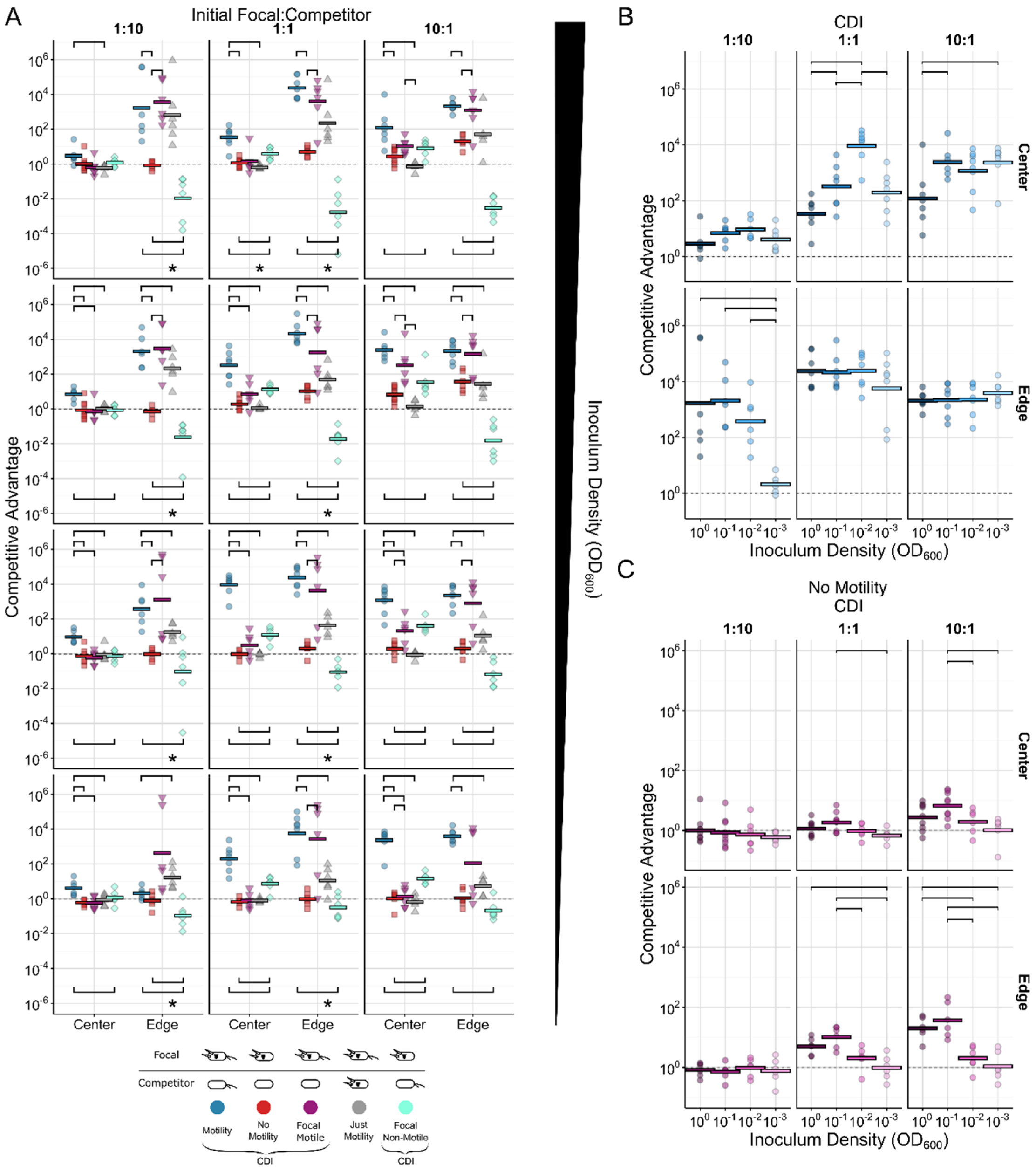

Figure S8 | Motility enhances the competitive advantage provided by CDI at the center and edge of colonies regardless of the initial density and attacker frequency. Colony competitions between wild-type or twitching motility deficient *P. aeruginosa* PAO1 and CDI-sensitive mutants were inoculated with different initial densities (OD_{600}) and ratios of attacker to sensitive cells. Quantification of the outcome of colony competitions picked at either the colony center or colony edge (**A**) reveal a consistently large advantage for motile attackers. Quantification of motile competitions from a range of inoculum densities and attacker:sensitive ratios (**B**) demonstrate a non-monotonic relationship between inoculum density and competitive advantage for 1:1 competitions at the colony center. Corresponding competitions using non-motile strains (**C**) do not show this effect. Competitive advantage is calculated as the fold-change in ratio of attacker:sensitive cells (as counted from sampling, plating and counting colony forming units) from the beginning to end of the experiment. Lines indicate the mean of replicates ($n \geq 6$). Top brackets indicate a significant difference between densities (one-sided Welch's t-test, $p < 0.05$, Benjamini-Hochberg MHT corrected 0.95).

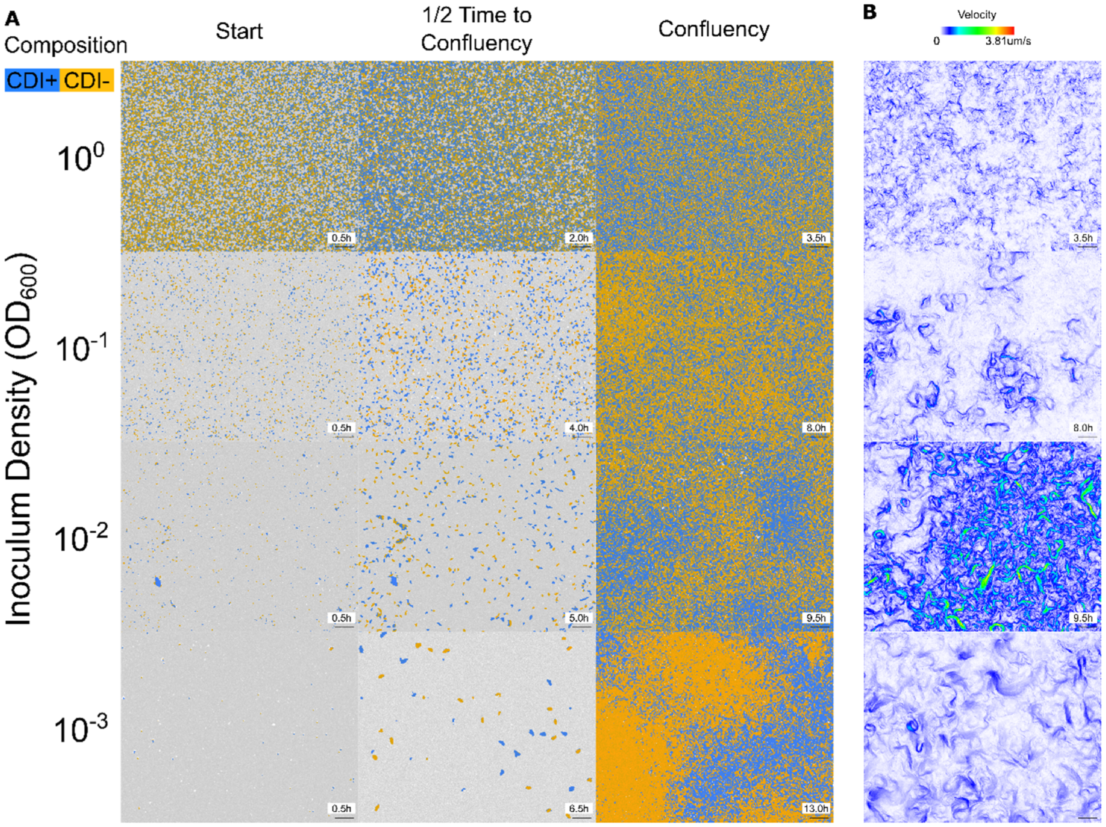

Figure S9 | Full field of view images of timelapse and fluorescence microscopy showing differences of strain mixing and motility in colonies inoculated at different densities. Colony competitions (1:1) between wild-type and CDI sensitive mutants were inoculated at different initial densities (OD_{600}) and imaged over time. Every 0.5 h after inoculation, a 1 min brightfield video was taken (0.5 frames s^{-1}) along with a single fluorescent snapshot in the YFP and mScarlet channels. Representative snapshots of colonies at the first time point ("Start", 0.5 h), the time when the surface was completely covered by cells ("Confluency", variable times), and halfway between the two timepoints ("1/2 Time to Confluency", variable times) (**A**) reveal increasing surface coverage with time, as well as the changing spatial distribution of wild-type attacker (blue) and CDI-sensitve (orange) strains. The velocity fields of colonies at confluency (**B**) also suggest an inverse relationship between inoculum density and cell motility. Images have been thresholded for display.

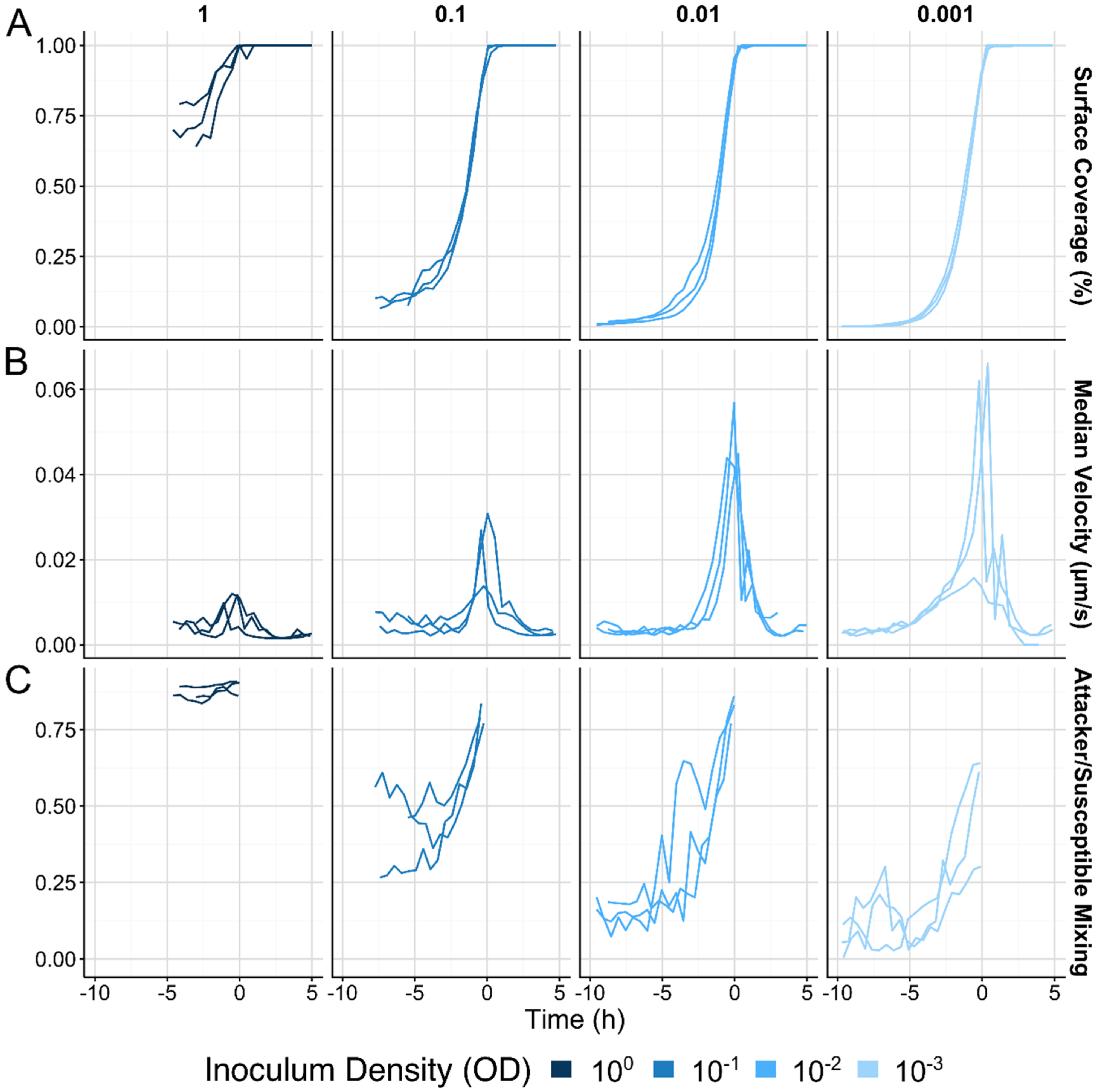

Figure S10 | Individual replicate measurements of surface coverage, velocity and strain mixing from timelapse and fluorescent microscopy. Individual replicates of colony competitions tracked in Figure 3. (**A**) percent area covered by cells, (**B**) median cell velocity magnitude and (**C**) extent of genotypic mixing between strains. Timecourses have been centered around the confluency time for each individual replicate.

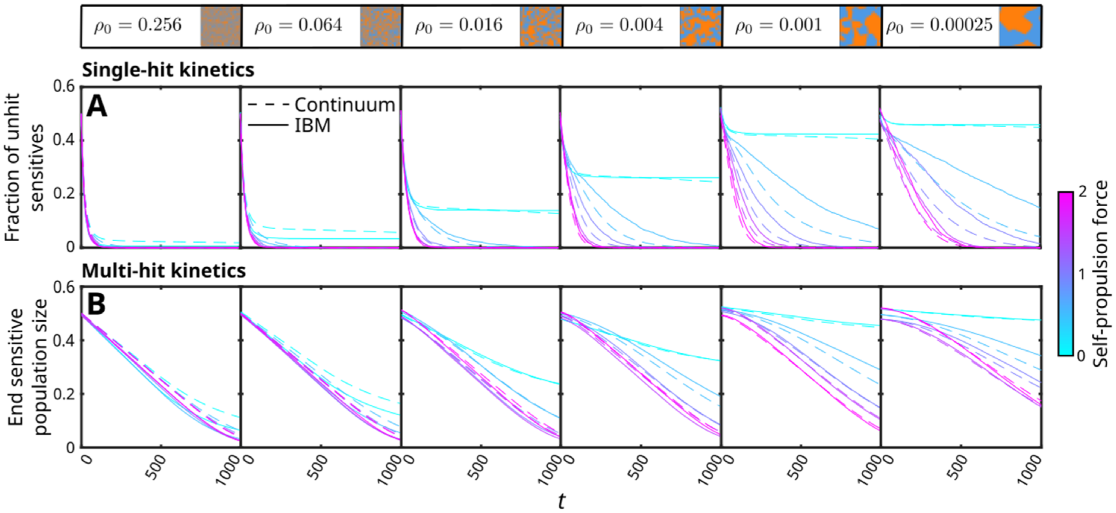

Figure S11 | Matching between the IBM and the continuum model is robust to changes in inoculum ratio and toxin potency. In our competition experiments, we consider colonies in which the attacker:sensitive inoculum ratio is 1:1 and the attacker's CDI toxin acts to slow down the growth of sensitives, rather than killing cells directly. To ensure that our matching between the two theoretical models was still effective with these changes, we performed additional simulations under these assumptions. In (**A**), we show the fraction of all cells that are sensitives that have not been hit as a function of time, equivalent to Fig. 1F but with a 1:1 rather than 1:9 inoculum ratio. This illustrates the killing dynamics under the assumption that one hit is sufficient to induce cell death. To simulate toxins that instead slow down growth with an accumulating impact with increasing numbers of hits, we introduce a toxin efficacy parameter $\xi = 0.02$ that represents the fractional slowdown in sensitive growth induced by each hit. In (**B**), we show the result of reducing the sensitive population size by the number of hits accumulated by each cell multiplied by ξ, which we take as a proxy for the final sensitive population size at the end of a competition experiment (Supplementary Note 4). Different seeding densities ρ_0 are indicated above each pair of plots, along with images of example continuum model starting states.

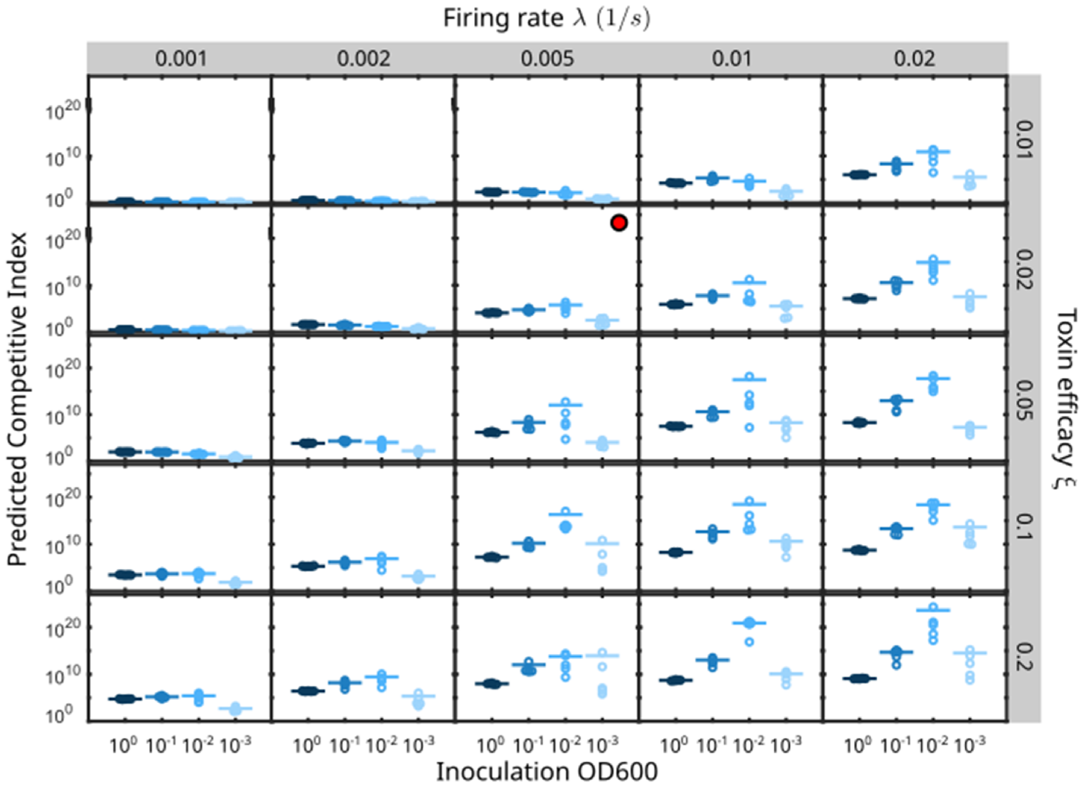

Figure S12 | The predicted non-monotonic relationship between inoculation density and intoxification efficiency is robust to variations in CDI firing rate and toxin potency. To test the impact of variations of our unconstrained parameters λ and ξ on our experimentally parameterized simulations (Fig. 4D-G), we ran a parameter sweep over these parameters while keeping all other factors fixed. Shown are the predicted competitive index of the attacker strain for *n*=5 simulations for each parameter combination, formatted equivalently to Fig. 4G. The combination of parameter values used in Fig. 4 is indicated with the red circle.
